# The use of dual biologic therapy for the management of recalcitrant psoriasis

**DOI:** 10.1016/j.jdcr.2024.02.036

**Published:** 2024-03-22

**Authors:** Paula Finnegan, Michelle Murphy, John Bourke

**Affiliations:** Department of Dermatology, South Infirmary Victoria University Hospital, Cork, Ireland

**Keywords:** dual biologic therapy, psoriasis, psoriatic arthritis

## Introduction

Biologic agents have transformed the management of several immune-mediated dermatologic conditions, leading to significant improvements in quality of life for several diseases including psoriasis, with high efficacy and a favorable safety profile reported.^1^ They are engineered monoclonal antibodies and fusion proteins that target specific proinflammatory mediators such as cytokines or cytokine receptors in the inflammatory pathway.^2^ They are often combined with nonbiologic treatment modalities, such as phototherapy or methotrexate, to achieve higher and quicker rates of skin clearance in the former, or improved joint symptoms in the latter.^1^ However, there are some complex patients, especially those suffering from concomitant psoriatic arthritis, that remain refractory to biologic monotherapy, or to the combination of a biologic agent with an oral systemic agent such as methotrexate.^1^^,^^3^ In these cases, dual biologic therapy may be considered as a treatment option.^3^ Dual biologic therapy is also considered in cases where patients have other comorbidities which limit the use of certain treatments, eg, inflammatory bowel disease and interleukin (IL)-17 inhibitors.^1^ This approach of combination biologic therapy for dermatology patients has been rarely reported in the literature. We report a case of a patient with psoriasis and psoriatic arthritis successfully treated with dual biologic therapy (adalimumab and guselkumab).

## Case report

We present a 68-year-old White woman with a 40-year history of psoriasis and distal psoriatic arthritis. Past medical history included active smoking status, hepatic steatosis, and type 2 diabetes mellitus. Previously failed treatment approaches included methotrexate, adalimumab, ustekinumab, secukinumab, brodalimumab, apremilast, ixekizumab, risankizumab, and filgotinib. Methotrexate was stopped due to worsening liver function tests. Her disease remained recalcitrant to all other trialed treatment modalities. She presented with a severe flare of psoriasis as well as a superimposed lower limb cellulitis. She was admitted for intravenous antibiotics. Following long discussion with the patient and rheumatology, the decision was made to commence dual biologic treatment in the form of the anti–tumor necrosis factor (TNF) agent adalimumab 80 mg once weekly, and the anti–IL-23 agent guselkumab 100 mg every 8 weeks. Her skin rapidly improved with reduced erythema and improved symptoms. She remains stable 6 months later without need for further hospitalization.

## Discussion

There are a wide variety of treatment options available for psoriasis including topical agents, phototherapy, methotrexate, cyclosporin, apremilast, acitretin, and biologic agents.^1^^,^^2^ Combination biologic therapy has been rarely reported for the treatment of psoriasis (Table I).^2^, ^3^, ^4^, ^5^, ^6^, ^7^, ^8^, ^9^, ^10^, ^11^, ^12^, ^13^ The potential rationale for this approach in patients with psoriasis can be understood by examining the pathophysiology of the condition.^3^ A chronic inflammatory immune-mediated condition, there are several other proinflammatory mediators involved in psoriasis, including IL-17, interferon gamma, TNF-alfa, and IL-23.^3^^,^^4^ Psoriasis is considered a systemic disease, where skin is just 1 of the many organs involved.^1^ Although IL-17 predominates in the development of psoriasis and endothelial cell damage, TNF-alfa is a major driver of the cutaneous manifestations of psoriasis, inducing epidermal hyperplasia, but also synovitis and enthesitis leading to joint symptoms.^5^ IL-17 causes dysregulation of T helper 17 cells and hyperresponsiveness.^3^ Given this multistep pathophysiology, it has been hypothesized that different organs (eg, skin and joints) may respond differently to the same treatment due to differing immunopathogenesis.^6^ Attempting to block several of these mediators rather than one in isolation may lead to better disease control.^5^ In addition to this, it has been suggested that neutralizing antibodies that target their prescribed monoclonal antibody medication may develop in some patients with psoriasis, leading to reduced treatment efficacy.^2^

**Table I:** Previous reports on combination biologic treatment in dermatology patients

Study (year)	Indication	Combination biologic
Adişen et al^10^ (2008)	Psoriatic arthritis	Efalizumab and etanercept
Babalola et al^4^ (2016)	Severe plaque psoriasis and psoriatic arthritis	Etanercept and ustekinumab
Cuchacovich et al^5^ (2012)	Psoriasis and psoriatic arthritis	Ustekinumab and etanercept; ustekinumab and adalimumab
Gniadecki et al^6^ (2016)	Psoriasis and psoriatic arthritis; psoriasis and psoriatic arthritis; psoriasis and psoriatic arthritis; psoriasis and psoriatic arthritis	Ustekinumab and etanercept; Ustekinumab and etanercept; ustekinumab and adalimumab, then ustekinumab and golimumab; ustekinumab and adalimumab, then ustekinumab and certolizumab
Hamilton^11^ (2008)	Psoriatic arthritis (*n* = 20)	Efalizumab and etanercept or infliximab
Hanna et al^3^ (2022)	Chronic plaque psoriasis and severe psoriatic arthritis	Risankizumab (IL-23 inhibitor) and golimumab (TNF-alfa inhibitor)
Heinecke et al^12^ (2013)	Psoriasis and psoriatic arthritis; psoriasis and psoriatic arthritis	Ustekinumab and etanercept; ustekinumab and etanercept, then ustekinumab and adalimumab
Kitamura et al^8^ (2009)	Psoriatic arthritis	Efalizumab and etanercept
Lowes et al^13^ (2005)	Psoriasis	Efalizumab and infliximab
Rathod et al^2^ (2019)	Psoriasis and psoriatic arthritis	Guselkumab and adalimumab
Thibodeaux et al^7^ (2019)	Psoriasis and psoriatic arthritis	Ustekinumab and etanercept, then secukinumab and etanercept, then guselkumab and etanercept
Torre et al^9^ (2017)	Palmoplantar pustulosis	Ustekinumab and adalimumab

*IL*, Interleukin; *TNF*, tumor necrosis factor.

There have been concerns raised about the potential increased risk of infection in patients on dual biologic therapy.^3^^,^^7^ In Gniadecki et al’s^6^ long-term case series observational study, they noted an increased frequency of infections including herpes zoster virus and retrotonsillar abscess. Kitamura et al^8^ report a case of tuberculosis in a patient on efalizumab and etanercept. In terms of other potential adverse effects, Babalola et al^4^ reported a cardiovascular event in a patient receiving dual biologic therapy (etanercept and ustekinumab), but they proposed that the event could not be definitively attributed to the treatment regime. Another potential factor to consider in dual biologic therapy is that of cost-effectiveness—although biologics are noted for their efficacy, concerns have been raised about the potential impact these medications will have on health care systems globally.^2^^,^^9^

In summary, we report a case of recalcitrant psoriasis and psoriatic arthritis where the use of dual biologic therapy resulted in successful control of dermatologic disease. Informed consent was obtained from the patient before initiation, and a multidisciplinary approach was used, discussing the risks and benefits before treatment induction. There were significant improvements in disease control and patient quality of life, with reduced hospital admissions, and improved activities of daily living. Dual biologic therapy may be an option for patients with disease that remains recalcitrant to biologic monotherapy. Further studies are needed to evaluate the safety, efficacy, and cost-effectiveness of this approach long-term.

## Conflicts of interest

None disclosed.
